# Thyroid Nodules in Children: A Single Institution's Experience

**DOI:** 10.1155/2011/974125

**Published:** 2011-10-06

**Authors:** Nini Khozeimeh, Cynthia Gingalewski

**Affiliations:** Department of Pediatric Surgery, Children's National Medical Center, 111 Michigan Avenue NW, Washington, DC 20010, USA

## Abstract

Thyroid nodules in children are uncommon but often present an increased risk of malignancy in comparison to their adult counterpart. Multiple diagnostic modalities are frequently employed to characterize these nodules including ultrasound, radionuclide scans, fine needle aspiration (FNA), thyroid function tests, and evaluation of patient demographics. We chose to evaluate if any of these modalities influence treatment or signify a tendency for a nodule to represent a malignant lesion. A retrospective review of patients <21 years of age who underwent partial or total thyroidectomy from 2004 to 2009 was performed (IRB no. 4695). Other than an FNA indicating a malignancy, there does not appear to be any value to extensive preoperative imaging, nor can patient risk be stratified based upon age. We conclude that there is minimal utility in an extensive preoperative workup in a child with a thyroid nodule.

## 1. Introduction

Thyroid cancer is the most common endocrine malignancy in pediatric patients [1–3]. However, thyroid nodules in children are only found in 3.7% of healthy children aged 11–18 years old [4]. Compared to adults, these nodules have an increased risk of being malignant (16% in children versus 5% in adults) [4–6]. Thyroid cancer in children is also unusual in that it often presents with advanced disease including lymph node involvement and lung metastasis as compared to their adult counterpart [5, 7–9]. 

Multiple modalities are frequently employed in an attempt to characterize thyroid nodules including ultrasound, radionuclide scans, fine needle aspiration (FNA), thyroid function tests, and evaluating patient demographics. In the adult population these results are frequently used to determine those patients who do not require thyroidectomy. Although these tests are also performed in the pediatric population, these patients frequently undergo partial or total thyroidectomy for diagnosis

The aim of this paper is to explore the utility of radiographic imaging and preoperative fine needle aspiration (FNA) in the evaluation of pediatric thyroid nodules, more specifically, to determine if any of these modalities identifies those thyroid nodules that are at high risk of harboring a malignancy.

## 2. Methods

A retrospective chart review was performed (2004–2009) at a tertiary medical center with a 100% pediatric patient population (IRB approval no. 4695). Fifty patients <21 years of age were identified who underwent partial or total thyroidectomy. Five patients with a family history of multiple endocrine neoplasia (MEN) were excluded, giving a total of 45 patients evaluated in the present study. Patient demographics, preoperative workup, type of procedure performed, and pathology results were reviewed. Calculations were performed using a 2-tailed Student's *t*-test.

## 3. Results

### 3.1. Demographics

Forty-five patients underwent either partial or total thyroidectomy; the majority of patients were female (*n* = 41). There was no difference in the median age at presentation for malignant and benign lesions 15 years (range of 7–19 years) versus 14 years (range of 8–21 years) (*P* = .88), respectively. None of the patients identified had a history of previous neck irradiation. Patient demographics and tumor characteristics are summarized in Table 1.

### 3.2. Preoperative Workup

Ninety-one percent of patients underwent preoperative imaging with ultrasound (*n* = 31), CT scan (*n* = 4), I^123^ scan (*n* = 2), or ultrasound and a second modality (*n* = 4). Of the 4 patients who had no preoperative imaging, three patients underwent thyroidectomy for a symptomatic, enlarging multinodular goiter. None of these patients were found to have malignancy. There were no herald radiologic findings to distinguish a lesion as malignant. Several nodules (*n* = 12) were found to have increased vascularity concerning malignancy; however, these nodules were found to be both malignant (*n* = 3) and benign (*n* = 9). FNA was performed in forty-nine percent of patients (*n* = 22). Of the nine patients found to have malignancy, five underwent preoperative FNA. Four FNAs were interpreted as malignant returning as papillary carcinoma. One FNA was interpreted as a follicular lesion but returned multifocal papillary carcinoma on final pathologic evaluation. This yields a sensitivity of 80% and specificity of 100%.

### 3.3. Disease Characteristics

Nine out of forty-five patients were identified with papillary carcinoma on pathologic examination (20%). Cervical lymph node involvement was identified in 56% percent of patients (*n* = 5) at the time of thyroidectomy. No patient had evidence of pulmonary metastases. There was no significant difference in the average size of the nodules in patients with malignant or benign disease (2.7 cm and 2.9 cm, resp., *P* = .63).

### 3.4. Surgical Procedure

Total thyroidectomy was performed as the initial procedure in those patients identified with malignancy by FNA (*n* = 4). Two patients underwent completion thyroidectomy after pathologic examination of their thyroid lobe revealed papillary carcinoma. Both of these patients did not undergo preoperative FNA. Both patients underwent preoperative imaging; the first patient with an ultrasound that showed a vascular nodule, while the second patient had a CT scan that showed a small nodule. In addition, one patient was diagnosed with papillary carcinoma on an intraoperative frozen section and had a completion thyroidectomy at that time. One patient had an incidentally found micropapillary carcinoma in the setting of Grave's disease, and one patient with a multinodular goiter had a preoperative FNA showing follicular cells, and the final pathologic diagnosis was papillary carcinoma. These patients did have preoperative imaging that showed nodules. 

Of those patients with benign disease (*n* = 36), 12 (33%) underwent a total thyroidectomy for either an enlarging multinodular goiter or Grave's disease refractory to medical treatment. Twenty-four patients underwent lobectomy. Sixteen underwent a right lobectomy (45%), and 8 had a left lobectomy (22%).

Hypocalcemia was the most common postoperative complication, occurring in 8 patients (38%). Half of these patients (*n* = 4) experienced symptoms with tingling or paresthesias, whereas the other half (*n* = 4) were identified with hypocalcemia on routine postoperative labs measurements. All patients were managed with supplemental oral calcium, and none experienced permanent hypocalcemia/hypoparathyroidism. All patients had normalization of their calcium levels within 3 months postoperatively. There were no injuries to the recurrent laryngeal nerve.

The average length of stay was similar for all patients after total thyroidectomy: 1.46 days for those with malignancy (range 1–5 days) and 1.53 days for benign pathology (range 1–8 days). All patients undergoing a thyroid lobectomy were discharged after a 23-hour observation period.

## 4. Discussion

Thyroid nodules in children represent an uncommon entity but carry a greater risk for malignancy than in their adult counterpart. In the pediatric population, an extensive preoperative workup of a thyroid nodule is not necessary. This should be limited to an ultrasound examination to determine that if indeed the nodule is within the thyroid gland. If feasible, given the patients age and anxiety level, a FNA should be performed. In those patients who have an inadequate FNA, or FNA is not performed, surgical excision should be performed for diagnosis. Total thyroidectomy remains the procedure of choice for those lesions identified preoperatively as cancer, while lobectomy should be employed for those lesions in which the diagnosis is uncertain [10–14]. This facilitates the use of I^131^ therapy postoperatively as well as the ability to monitor thyroglobulin levels after treatment for recurrent disease. Overall, thyroid cancer has a very good prognosis with a 98.8% survival at 10 years in children [2, 7].

The female predominance of thyroid nodules noted in our cohort has been previously reported in the literature and is likely secondary to the estrogen sensitivity of the thyroid gland [15–18]. 

In our cohort, patient age and radiologic imaging bears no impact in determining whether a nodule was malignant or benign. Increased intranodular vascularity is an ultrasound characteristic that has been suggested as an indicator of malignancy [19, 20]. In our group, patients were found to have intranodular vascularity suggestive of malignancy (*n* = 12) however, the lesions returned both benign (*n* = 9) and malignant (*n* = 3). Average nodule size also did not signify a predisposition for malignancy. 

Fine needle aspiration and cytology evaluation in the pediatric patient is controversial [5, 21–25]. FNA is useful for preoperative planning if the lesion is identified as malignant. But the lesion may be identified as benign, indeterminate, or follicular. Follicular lesions cannot be differentiated as malignant or benign because of the inability of FNA to assess capsular invasion. If the lesion is identified as malignant, the patient should undergo a total thyroidectomy at initial operation versus an initial lobectomy when indeterminate or benign. If FNA identifies the nodule as benign or a cyst, a patient can be followed if the lesion is small and avoid operative intervention. In adults, the accuracy of FNA can reach 97%, while the pediatric population accuracy only reaches 90% [5, 8, 21, 23, 26]. Other limitations of FNA include sampling error, experience of the cytopathologist, or need for sedation in younger children.

Postoperative complications of hypocalcemia and recurrent laryngeal nerve injury are a great cause of concern in patients undergoing total thyroidectomy. The most common complication following total thyroidectomy is hypocalcemia. Hypocalcemia is largely due to incidental stunning or removal of the parathyroid glands, which may be embedded in the thyroid gland [7, 27]. The parathyroid glands may also be devascularized during thyroid gland dissection. The resultant hypocalcemia can be either transient or permanent.

Permanent hypocalcemia was not encountered in our patient population. Another risk of total thyroidectomy is recurrent laryngeal nerve injury (RLN), resulting in hoarseness, dysphagia, and respiratory failure if a bilateral injury occurs. Permanent RLN after total thyroidectomy has been reported as 1% in the literature [28, 29]. We report no instances of RLN injury in our group.

In our retrospective review, we report no instances of major complications, such as RLN injury or permanent hypocalcemia, following total thyroidectomy. Transient hypocalcemia is considered to be of moderate risk and is readily managed with oral calcium supplementation.

In conclusion, there does not seem to be any component found in the preoperative evaluation of a pediatric thyroid nodule, with the exception of a positive FNA to indicate that a lesion is malignant or benign. Our experience suggests that patients cannot be risk stratified based on age, size of the thyroid lesion, or characteristics of the lesion by all radiographic imaging techniques. In addition, postoperative risk to patients undergoing lobectomy or total thyroidectomy remains low and predominantly consists of transient hypocalcemia. At our institution we have found minimal utility of an extensive preoperative workup in a child with a thyroid nodule. CT scans have not been shown to be sensitive or specific for determining whether a lesion is cancerous or benign. Therefore, the lifelong risk of cancer associated with CT is not justified (lifetime cancer risk associated with CT is estimated to be 2/1000 to 3/1000 in children under age 15). We advocate ultrasound evaluation to determine an intrathyroidal location, an FNA if feasible, and then either total thyroidectomy for those with malignancy identified on cytology or lobectomy for those whose diagnosis is uncertain. 

## Figures and Tables

**Table 1:** Demographic, tumor, and clinical characteristics. Thyroid nodules were identified as malignant or benign based on pathologic characteristics. These nodules were then characterized based on patient age, gender, and preoperative workup including imaging or FNA.

	Entire cohort *n* = 45	
	Malignant	Benign
Total patients	9	36
Gender		
Female	8	33
Male	1	3
Average age at diagnosis (years)	14.1	14.2
Average size of nodule (cm)	2.7	2.9
FNA		
Yes	5	17
No	4	19
Radiologic imaging		
Ultrasound	5	26
CT scan	3	1
Nuclear medicine	0	2
Ultrasound and 2nd modality	1	3
